# Prevalence and Sources of Duplicate Information in the Electronic Medical Record

**DOI:** 10.1001/jamanetworkopen.2022.33348

**Published:** 2022-09-26

**Authors:** Jackson Steinkamp, Jacob J. Kantrowitz, Subha Airan-Javia

**Affiliations:** 1 Department of Medicine, Perelman School of Medicine at the University of Pennsylvania, Philadelphia; 2 River Records, LLC, Jamaica Plain, Massachusetts; 3 TrekIT Health, Inc, CareAlign, Philadelphia, Pennsylvania

## Abstract

**Importance:**

Duplicated text is a well-documented hazard in electronic medical records (EMRs), leading to wasted clinician time, medical error, and burnout. This study hypothesizes that text duplication is prevalent and increases with time and EMR size and that duplicate information is shared across authors.

**Objective:**

To examine the prevalence and scope of duplication behavior in clinical notes from a large academic health system and the factors associated with duplication.

**Design, Setting, and Participants:**

This retrospective, cross-sectional analysis of note length and content duplication rates used a set of 10 adjacent word tokens (ie, a 10-gram) sliding-window approach to identify spans of text duplicated exactly from earlier notes in a patient’s record for all inpatient and outpatient notes written within the University of Pennsylvania Health System from January 1, 2015, through December 31, 2020. Text duplicated from a different author vs text duplicated from the same author was quantified. Furthermore, novel text and duplicated text per author for various note types and author types, as well as per patient record by number of notes in the record, were quantified. Information scatter, another documentation hazard, was defined as the inverse of novel text per note, and the association between information duplication and information scatter was graphed. Data analysis was performed from January to March 2022.

**Main Outcomes and Measures:**

Total, novel, and duplicate text by note type and note author were determined, as were the mean intra-author and inter-author duplication per note by type and author.

**Results:**

There were a total of 104 456 653 notes for 1 960 689 unique patients consisting of 32 991 489 889 words; 50.1% of the total text in the record (16 523 851 210 words) was duplicated from prior text written about the same patient. The duplication fraction increased year-over-year, from 33.0% for notes written in 2015 to 54.2% for notes written in 2020. Of the text duplicated, 54.1% came from text written by the same author, whereas 45.9% was duplicated from a different author. Records with more notes had more total duplicate text, approaching 60%. Note types with high information scatter tended to have low information overload, and vice versa, suggesting a trade-off between these 2 hazards under the current documentation paradigm.

**Conclusions and Relevance:**

Duplicate text casts doubt on the veracity of all information in the medical record, making it difficult to find and verify information in day-to-day clinical work. The findings of this cross-sectional study suggest that text duplication is a systemic hazard, requiring systemic interventions to fix, and simple solutions such as banning copy-paste may have unintended consequences, such as worsening information scatter. The note paradigm should be further examined as a major cause of duplication and scatter, and alternative paradigms should be evaluated.

## Key Points

**Question:**

How much duplicate content is present in electronic medical records, where does it come from, and why is it there?

**Findings:**

In this cross-sectional analysis of 104 456 653 routinely generated clinical notes, 16 523 851 210 words (50.1% of the total count of 32 991 489 889 words) were duplicated from prior documentation. Duplicate content was prevalent in notes written by physicians at all levels of training, nurses, and therapists and was evenly divided between intra-author and inter-author duplication.

**Meaning:**

The prevalence of information duplication in electronic medical records suggests that it is an adaptive behavior requiring further investigation so that improved documentation systems can be developed.

## Teaser

This cross-sectional study examines the prevalence and scope of duplication behavior in clinical notes from a large academic health system and the factors associated with duplication.

## Introduction

### The Note Paradigm

Text documentation within electronic medical records (EMRs) in the US largely uses the note paradigm for information organization. This paradigm and its attendant software were intended to re-create the functionality from the era of the paper-based chart.^1,2^ The paradigm assumes that (1) each encounter with a patient requires a separate document to be created, and (2) different authors should document in entirely separate documents. As such, text documentation in a patient record is organized primarily by time and author*,* rather than clinical topic, although text within an individual note is often arranged by topic.

### Information Hazards

Adherence to the note paradigm leaves little room for modern software advances, such as document editing, collaborative documentation, or version history tracking, and contributes to 2 major documentation hazards common in modern EMRs: information overload and information scatter.^3,4^ Information overload refers to the difficulty of finding relevant information because there is too much information in the EMR.^4^ Information overload includes information duplication, when the same content is replicated throughout the EMR by copy-pasting, templating, and summarizing. Duplicated information wastes clinician time on tedious fact searching and confirmation, generates inaccurate documentation, obfuscates the original source of text, and ultimately leads to medical errors.^5,6,7,8,9,10,11,12,13^

Information scatter refers to the difficulty of finding and synthesizing information because it is fragmented across numerous locations^4^ and leads to wasted time retrieving data, or worse, missed information because clinicians lack time to adequately search the EMR. Scatter has been a problem for medical records since the late 19th century, when each service would keep separate, serially generated, and bound records.^14^ However, scatter was balanced by the efficiency of the paper-based system (eg, a single envelope could hold an entire chart), and notes could be as succinct as a presenting symptom, diagnosis, and treatment. Notably, when 2 encounters were close in time, physicians would know that relevant details would be previously documented and that there would be no point duplicating the same information at each encounter.^1^

### The Roots of Duplication

Documentation behaviors are influenced by the assumptions underlying electronic documentation systems, as well as related billing and legal requirements. Prior research^15^ has found as much as 58% of physician note content is duplicated from the author’s last note and that note length and redundancy increase with time. Given the pervasiveness of information duplication, this behavior may be a rational and adaptive response to poorly designed documentation paradigms and software interfaces. In particular, duplication may be incentivized by the note paradigm, the time*-*based and author*-*based organization of modern EMRs. No study to our knowledge has described the proportions of duplicate content attributable to the same author as compared with other authors. Furthermore, no study has framed the issue of duplication around the note-based documentation paradigm.

### Our Study

In this study, we quantified the total text in the EMR and how much of it is duplicated. We characterized how duplication fraction changed with note type and author type. In addition, we hypothesized that information duplication would increase with increasing patient record size and with time. Because the note paradigm requires new notes to be created for each encounter (including each day of an inpatient stay), we analyzed the relative proportion of duplication that occurs in note types with different update frequencies (eg, a new inpatient progress note is created every 24 hours, but outpatient progress notes are written at greater intervals). We hypothesized that notes written at higher update frequencies (eg, inpatient progress notes) would have higher proportions of duplicated text. Furthermore, we compared duplication frequencies from the same author with duplication across authors to quantify whether (1) requiring new documents every time a single author sees the patient and (2) requiring different clinicians to use separate documents would affect duplication prevalence*.*

We analyzed more than 100 million clinical notes across all medical specialties over the course of 6 years, including notes written by all members of the clinical team. Previous work^7,15,16,17^ in this space has looked at much smaller^17^ or limited sets of notes (eg, only outpatient notes or only physician notes). Furthermore, ours is the first study, to our knowledge, to examine duplication behaviors in the light of the note paradigm and the incentives it creates.

## Methods

This is a retrospective, cross-sectional analysis of clinical documentation produced during routine health care operations. The study was approved by the University of Pennsylvania institutional review board and follows the Strengthening the Reporting of Observational Studies in Epidemiology (STROBE) reporting guideline. Obtaining consent from all participants was deemed infeasible and was waived by the institutional review board.

We analyzed a corpus of all electronic notes written within the Penn Medicine Health System over 6 years, from January 1, 2015, through December 31, 2020. We included all types of notes, clinical contexts, and author types from all adult patient encounters. Penn Medicine encompasses inpatient and outpatient practices, including 6 acute-care medical facilities and 2941 inpatient beds. There were 129 016 inpatient admissions, 337 712 ED visits, and 5 684 554 outpatient visits in 2021. During this time, the outpatient EMR was Epic (Epic Systems). The inpatient EMR was Sunrise Clinical Manager (Allscripts) from 2015 to 2017 and Epic thereafter.

### Statistical Analysis

 Data analysis was performed from January to March 2022. Analyses were conducted using the Python programming language version 3.9 (Python Software Foundation) and the Apache Spark engine version 3.2 (Apache Software Foundation) in a Databricks environment version 9.1 (Databricks) hosted on Azure (Microsoft).

First, we characterized the size of the corpus. Second, we quantified the fraction of duplicate text in each note. We defined duplicate text as any span of text that appears in a previous note for the same patient (regardless of note author). To identify duplicate text spans, we looked at each 10-gram (ie, set of 10 adjacent word tokens) in each note, and if that 10-gram occurred in a previous note or earlier in the same note, that span and its tokens were marked as duplicate. We advanced the 10-gram window 1 token at a time until all 10-grams were evaluated; if a word token appeared in at least 1 duplicate 10-gram window, it was considered duplicated (see eAppendix 1 in the Supplement for the code). Duplicate 10-grams are relatively unlikely to occur by chance without intentional duplication behavior (see eAppendix 2 in the Supplement for details and our reasoning for using the n-gram sliding window algorithm rather than sequence alignment methods). A word token refers to the smallest unit of meaning (ie, a word or semantically meaningful symbol). From here on, for legibility we will refer to a word token simply as a word.

We used this method to quantify the proportion of duplicated text vs novel (nonduplicated) text. Furthermore, we quantified text duplicated from prior notes written by the same author vs by other authors. For this analysis, the most recent note with the same text in it was presumed as the source of the duplication (see eAppendix 3 in the Supplement for a discussion of limitations of this method).

We also graphed the association between information duplication and information scatter for different note types. Intuitively, scatter is the inverse of information concentration (novel text per note). Therefore, our metric for scatter is the number of notes a clinician would have to read to get 500 words of novel text. For instance, a series of short notes is more scattered than a single note containing the same information in 1 place.

## Results

### Corpus

The corpus consisted of 104 456 653 notes for 1 960 689 unique patients, including 32 991 489 889 words or 192 618 267 371 characters of text. The median patient record length was 4285 words (range, 1-6 598 041 words) with a mean (SD) length of 16 826 (49 911) words. The median note length across all author types was 62 words (range, 1-116 964 words); the median physician-authored note length was 294 words (range, 1-116 964 words). See eTable 1 and eTable 2 in the Supplement for tabular breakdowns of text volume by note type and author type.

### Prevalence of Duplicate Content

**Figure 1.**
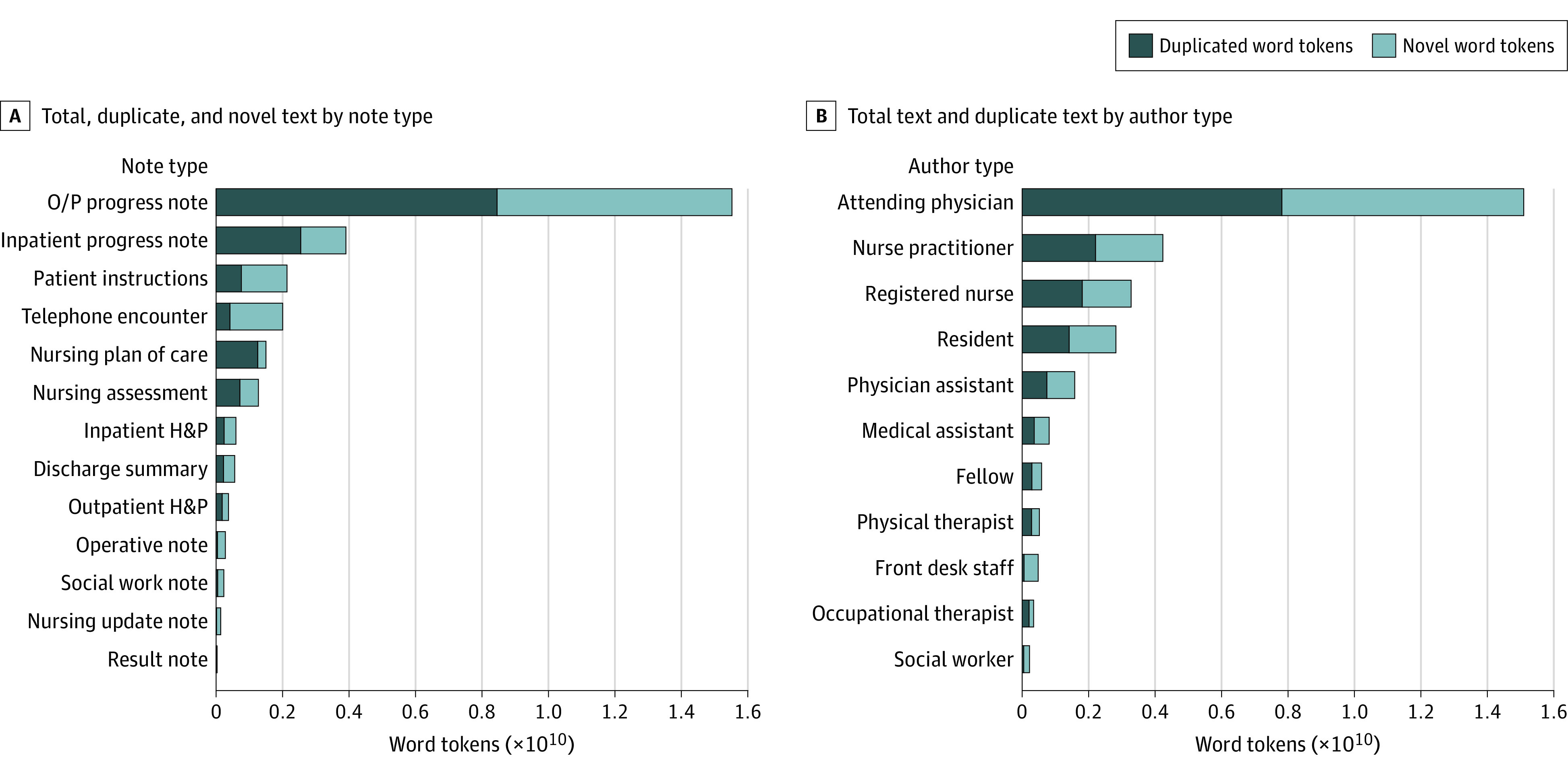
Prevalence of Duplicate Content by Note and Author Type Graphs show the total amount of text in the corpus grouped by note type (A) and author type (B), as well as the amount of novel (light blue) vs duplicate (dark blue) text in each grouping. Notes written by clinicians have larger proportions of duplicate text than those written by the front desk. All author and note types contain some duplicate content. Result note refers to outpatient notes documenting actions taken related to diagnostic studies; telephone encounter refers to notes documenting telephone encounters with a patient. H&P indicates history and physical; O/P, outpatient.

**Figure 2.**
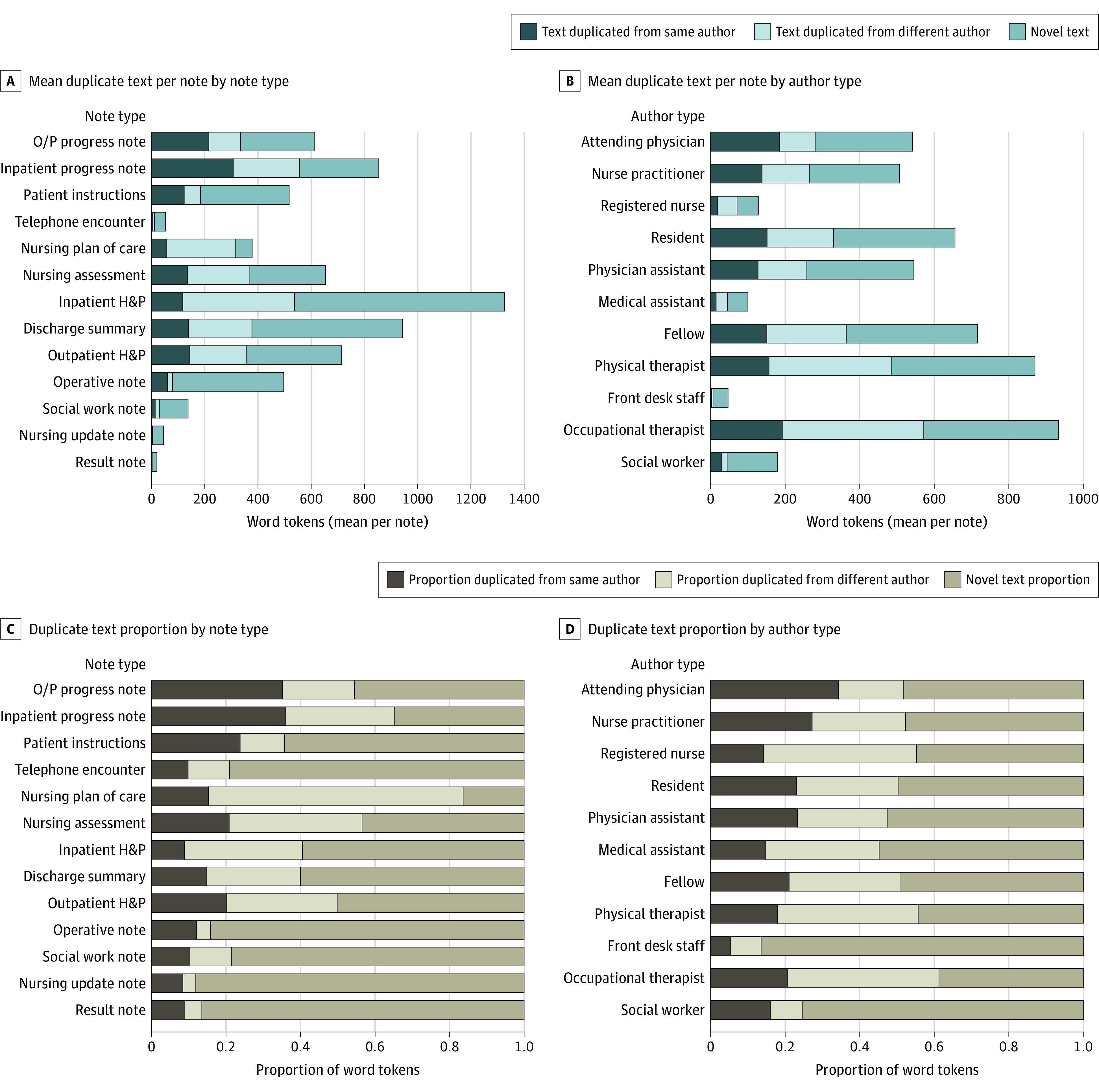
Information Sharing Across Authors Graphs show the median length of an individual note grouped by note type (A and C) and author type (B and D), with mean amounts of duplicate text by source (same vs different author). Note and author types are organized in the same order as Figure 1, for readability. H&P indicates history and physical; O/P, outpatient.

In total, 50.1% of total clinical text (16 523 851 210 words) was duplicated from previous text written about the same patient, whereas 49.9% (16 467 538 679 words) was novel text. This duplication was present in all note types and regardless of author type (Figures 1 and 2).

### Duplicate Content From Multiple Sources

We quantified the amount of text duplicated from notes by the same author vs notes written by a different author (Figure 2). Since our model frames the duplication problem in terms of (1) requiring new documents every time a single author sees the patient and (2) requiring different clinicians to use separate documents, this analysis facilitates a relative quantification of these 2 contributions. Text copied from a previous note by the same author reflects the first problem, and text copied from a different author reflects the second problem. Our analysis found that 54.1% of duplicate text came from a previous note by the same author (or earlier in the same note), and 45.9% came from a note by a different author.

The amount of duplicate text varied across different note types, with nursing plan of care notes and physician progress notes consisting of majority duplicate text (Figures 2A and 2C). These notes are required to be comprehensive while also having to be updated at least daily. Notes designed to document single events (ie, telephone encounter, result note, and operative note) had a smaller proportion of duplicated text (Figures 2B and 2D). Notes written by front desk staff and social workers were shorter (Figure 2B) and contained a lower proportion of duplicated text than notes written by physicians, nurses, and other clinicians; in particular, notes from physicians included 30% to 70% duplicate content (Figure 2D).

### Association of Increased Duplicate Content With Record Size and Time

**Figure 3.**
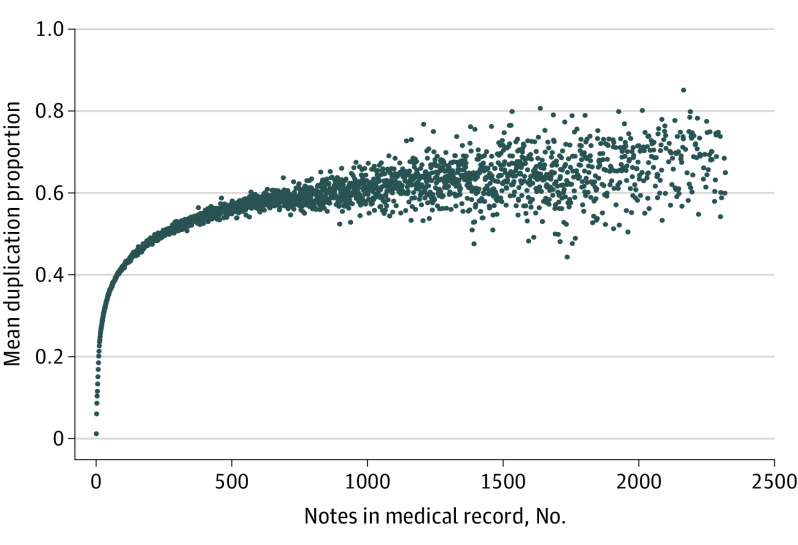
Proportion of Duplicate Text per Record by Size of Medical Record Each data point represents the mean duplication proportion for medical records of a particular length. The findings show that larger records contain more duplicate content.

Duplication rates were lower in records with a smaller number of notes (Figure 3). Duplication behavior tended to become more prevalent in larger records, although a sort of asymptotic plateau effect was observable. Patient records with more notes had more total duplicate text, approaching 60%.

**Figure 4.**
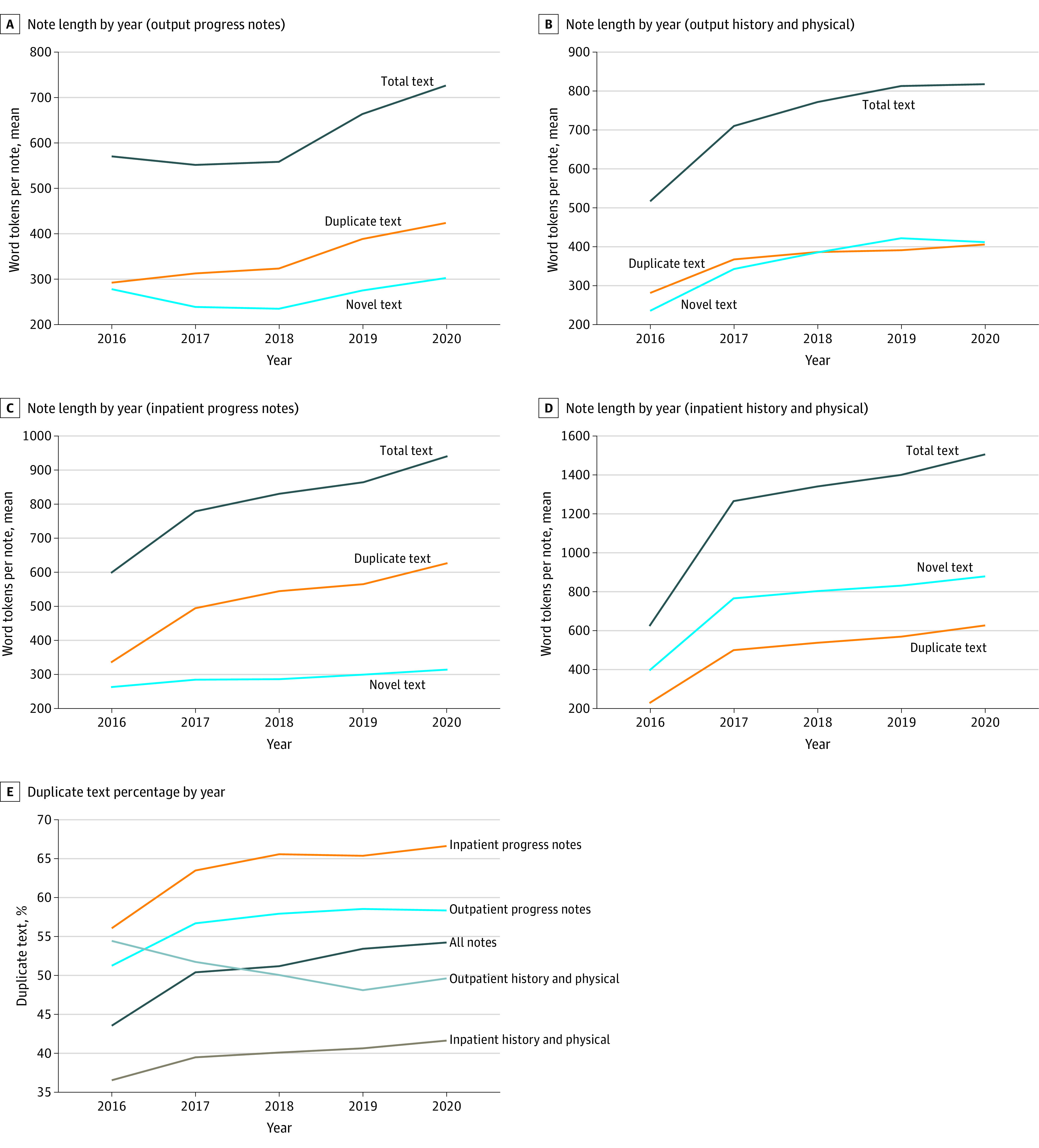
Changes in Note Length and Duplication Fraction Over Time Graphs show yearly averages of novel text, duplicate text, and total text per note for inpatient history and physical notes (A), inpatient progress notes (B), outpatient history and physical notes (C), and outpatient progress notes (D). Panel E shows percentages of duplicate text for all 4 note types on the same graph. Graphs show that note length and duplication fraction both increased over time.

We studied the proportion of duplicate text in notes over time (Figure 4) for 4 common note types (history and physical and progress notes in inpatient and outpatient settings). For all 4 note types, total note length increased steadily from 2015 to 2020. For inpatient and outpatient progress notes, duplicate text increased more than novel text over this period. The amount of duplicate text increased from 33.0% for notes written in 2015 to 54.2% in 2020. For history and physical notes, both duplicate and novel text increased.

### Information Duplication and Scatter Trade-off

Information overload and scatter are both hazards within the EMR. We plotted duplication (a quantifiable surrogate for overload) and scatter for different note types to investigate the association between these 2 documentation hazards. Information scatter, quantified as the number of notes required to produce 500 words of novel text, can be thought of as the opposite of information density. For instance, telephone encounter notes have, on average, 42 words of novel text per note; this would mean that a clinician reading the record would have to view approximately 10 separate notes to get 500 words of novel text, an extremely scattered set of notes requiring many clicks to navigate. On the other hand, inpatient history and physical notes have an average of 798 novel words per note, meaning it only takes 0.62 history and physical notes to find 500 words of novel text. Therefore, inpatient history and physical notes are much less scattered than telephone encounter notes under the current paradigm.

**Figure 5.**
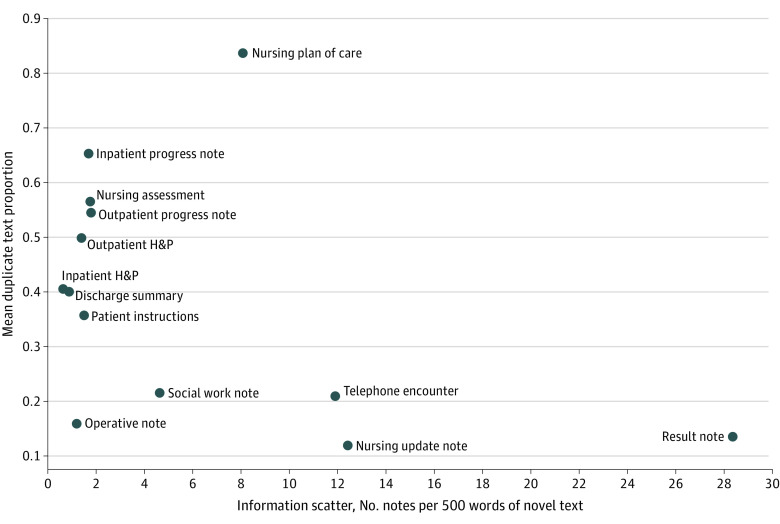
Trade-off Between Scatter and Duplication Both duplication and scatter are major hazards with the modern medical record. Notes with low scatter and low duplication represent the ideal note. In our corpus, most note types have either high scatter or high duplication but not both, suggesting a partial trade-off between duplication and scatter under the current documentation paradigm. H&P indicates history and physical.

In visualizing scatter vs duplication (Figure 5), 2 major clusters of note types emerge, illustrating a trade-off between scatter and duplication. Many note types with high mandated update frequencies (eg, progress notes or nursing assessment notes) have relatively low scatter and high duplication. Notes that document single events, such as telephone calls and nursing update notes, have low duplication but extremely high scatter. Operative notes have low scatter and low duplication; they are relatively lengthy accounts of a contained event (an operation) and are not regularly updated after writing.

## Discussion

In this cross-sectional study, which to our knowledge is the largest of its kind, we analyzed more than 100 million notes to characterize the prevalence of duplication in the EMR. More than half of all text was duplicated, more than 16.52 billion words in total. As of February 2022, the entirety of all English Wikipedia articles contained approximately 3.9 billion words^18^; our note corpus is approximately 8 times as large, more than half of which was duplicated. Duplicate content was prevalent in notes written by physicians at all levels of training, nurses, and therapists, and was evenly divided between intra-author and inter-author duplication. Physicians wrote the notes with the largest amounts of novel information, but also wrote the longest notes, and included 30% to 70% duplicate content, so their notes were comprehensive but repetitive. In addition, as the number of notes increases, the proportion of duplicate content increases and then plateaus. Given the ubiquity of duplication, this practice cannot be attributed to individual authors behaving badly, nor could it be safely banned without larger changes to underlying paradigms. Instead, our study suggests that duplication is a rational response of clinicians attempting to manage information in a documentation paradigm ill-suited to the task.

### Clinical Importance

Information overload and duplication are severe hazards for practicing clinicians. Finding the right information is no longer a matter of flipping through a paper chart; it is more akin to reading large portions of a book (the mean patient record has 56% of the word count of William Shakespeare’s longest written work, *Hamlet*).^19^ Our analysis shows that roughly half of text is directly duplicated, word for word, from elsewhere in the record, compounding the challenge of finding the right data to make appropriate clinical decisions. In the corpus described here, the median record length was 4285 words; therefore, 10 records is 42 850 words, which is 81 standard single-spaced pages of 500 words each. Thus, a physician seeing 10 patients in a day would be responsible for reviewing at least 85 pages of single-spaced text across 691 notes. The duplicated half of the content not only provides no new information, but also increases the time required for the reading clinician attempting to discern which information is accurate and timely vs false or irrelevant. Overworked clinicians may be disincentivized from reading such a bloated record, missing valuable clinical context not easily found elsewhere (eg, reasons for past diagnostic or therapeutic decisions), and leading to wasted time repeating past interventions or directly causing patient harm by missing findings requiring follow-up. Second, rampant duplication creates viral copies of errata that can spread through a record until they are impossible to correct because of the number of copies and the inability to mark information as erroneous.

The prevalence of duplicate content suggests that there is a subset of clinical information that remains true and relevant over time and, therefore, ideally, should remain visible in the EMR. However, with current documentation paradigms, copying old information to the latest note is the main way clinicians accomplish this goal in an EMR that few have time to read. Unfortunately, this itself worsens information overload, leading to a vicious cycle. We propose that documentation systems be redesigned to take advantage of the stability of shared information over time. Duplication across authors might be addressed using collaborative documentation systems, in which each individual or team does not require a completely separate document. Duplication across time might be addressed by building documentation systems that enable editing and version history functionality to track changes to a single dynamic document. This functionality allows updates to be made without requiring creation of a new document, while maintaining old documents for medicolegal purposes. Multiple such systems have been described and implemented in medical contexts, described as dynamic documentation or the wiki model,^3,20,21,22,23^ but have not yet been widely implemented.

Information scatter is another major documentation hazard that must be balanced against information duplication and overload. Information that should be stored in 1 place for effective synthesis (eg, the evolution of a chronic medical problem and its treatment over years) is instead stored across hundreds of separate notes.^4^ Under the current system, to keep relevant information about a patient in a single, up-to-date document (ie, minimize scatter), a note author will need to continuously create copies of old notes and add to them, rather than just editing old documents in place. This practice directly contributes to textual duplication.^3,22^ Therefore, we cannot treat duplication in isolation, as unilateral restrictions on copy-paste behavior may exacerbate information scatter. Our study provides some evidence for this trade-off in our analysis of note lengths and novel text. Telephone notes are short and mostly contain nonduplicated text; however, as a result, many notes are required to communicate the same amount of information (high scatter). On the other hand, physician inpatient progress notes are long and have large amounts of duplicate text (high duplication); however, most of the relevant information remains in a single, easily accessible document (decreased scatter). Further study is needed to elucidate this trade-off and to identify better metrics to assess information scatter. Administrators should be wary of simple solutions such as an outright ban on duplication; without addressing the clinical need to maintain information visibility, these solutions will only exacerbate other hazards. In future work, we plan to perform qualitative examinations of duplicated text to further characterize its types and sources (see eAppendix 4 in the Supplement).

### Limitations

This study has limitations that should be addressed. Our methods could capture exactly duplicated text only, not summarized or paraphrased duplicate information. For this reason, our study likely underestimates total duplicate text. In addition, our measurements of text duplicated from other authors may partially reflect shared clinical templates. Our metric for assessing duplication source is also imperfect (eAppendix 3 in the Supplement). Furthermore, our conclusions may not apply equally well to other health systems or EMRs, because our study was limited to a single health center and a single EMR. In particular, our health system has a high percentage of trainees, which may affect duplication behavior.

## Conclusions

Information duplication is not primarily a mistake made by individual clinicians; rather, given its ubiquity, duplication must be viewed as a result of the documentation system. That system is made up of specific EMR software, institutional documentation practices, and an underlying paradigm. The note paradigm for documentation should be further examined as a major cause of duplication and scatter, and alternative paradigms should be evaluated.

## Notes

Supplement. **eAppendix 1.** Code **eAppendix 2.** Comparison of Methodology to Prior Work **eAppendix 3.** Discussion of Limitations of Our Methods **eTable 1.** Median, Mean, and Total Note Length in Characters by Note Type **eTable 2.** Note Lengths by Provider Type, Sorted by Median Note Length **eAppendix 4.** The Utility of Shared Clinical Templates
