# Supplementary material for: Prevalence and Sources of Duplicate Information in the Electronic Medical Record
Source: JAMA Netw Open. 2022 Sep 26;5(9):e2233348. doi: 10.1001/jamanetworkopen.2022.33348 (PMC9513649; doi:10.1001/jamanetworkopen.2022.33348)
Supplement: Supplement. — eAppendix 1. Code eAppendix 2. Comparison of Methodology to Prior Work eAppendix 3. Discussion of Limitations of Our Methods eTable 1. Median, Mean, and Total Note Length in Characters by Note Type eTable 2. Note Lengths by Provider Type, Sorted by Median Note Length eAppendix 4. The Utility of Shared Clinical Templates [file jamanetwopen-e2233348-s001.pdf]

Supplemental Online Content

Steinkamp J, Kantrowitz JJ, Airan-Javia S. Prevalence and sources of duplicate information in the electronic medical record. *JAMA Netw Open*. 2022;5(9):e2233348. doi:10.1001/jamanetworkopen.2022.33348

eAppendix 1. Code
eAppendix 2. Comparison of Methodology to Prior Work
eAppendix 3. Discussion of Limitations of Our Methods
eTable 1. Median, Mean, and Total Note Length in Characters by Note Type
eTable 2. Note Lengths by Provider Type, Sorted by Median Note Length
eAppendix 4. The Utility of Shared Clinical Templates

This supplemental material has been provided by the authors to give readers additional information about their work.

© 2022 Steinkamp J et al. *JAMA Network Open.*

eAppendix 1. Code

Below is the code for our n-gram sliding window algorithm, as implemented using Python and Spark.

Code

```
from pyspark.sql.window import Window
import pyspark.sql.functions as F

from spacy.tokenizer import Tokenizer
from spacy.lang.en import English

# create spacy tokenizer
nlp = English()
tokenizer = Tokenizer(nlp.vocab)

# load notes and group them by MRN
notes = spark.read.parquet(***INSERT FILENAME HERE***)
structed = notes.withColumn('struct',F.struct("FULL_TEXT","NOTE_ID",”TIMESTAMP”))
grouped = structed.groupBy("mrn").agg(F.collect_list('struct').alias('notes'))

def ngrams(texts, n=2):
  ngs = []
  for text in texts:
    ngs += zip(*[text[i:] for i in range(n)])
  return ngs

# get all ranges
import itertools
def ranges(i):
    for a, b in itertools.groupby(enumerate(i), lambda pair: pair[1] - pair[0]):
        b = list(b)
        yield b[0][1], b[-1][1]



# count amount of copypasted text
def spark_cp_count(note_list):
  ng_size = 10
  all_ngs = {}
  out = []

  # sort each patient’s notes by note timestamp
  nl = sorted(note_list,key=lambda x: x['TIMESTAMP'])

  # for each note
  for note in nl:
    nid = note['NOTE_ID']

    # tokenize it and get the ngrams
```

© 2022 Steinkamp J et al. *JAMA Network Open.*

```
    doc = tokenizer(note['FULL_TEXT'])
    tokens = [i.text.strip() for i in doc]
    pos = [None for t in tokens]
    ngs = ngrams([tokens],n=ng_size)


    # if an n-gram has already been seen for this patient, mark all words within it as duplicated
    for idx,ng in enumerate(ngs):
      if ng in all_ngs:
        for i in range(idx,idx+splitter_gram):
          pos[i] = all_ngs[ng]
      all_ngs[ng] = note["NOTE_AUTH"]

    # sum up n-gram data for this note - how much is copy-pasted, how much is from same
author, etc.
    cp_spans = [(r[0],doc[r[0]:r[1]+1],True) for r in (ranges([i for i in range(len(pos)) if pos[i] is not
None]))]
    orig_spans = [(r[0],doc[r[0]:r[1]+1],False) for r in (ranges([i for i in range(len(pos)) if pos[i] is
None]))]
    total_length_char = sum([len(tup[1].text) for tup in cp_spans + orig_spans])
    total_length_tok = sum([len(tup[1]) for tup in cp_spans + orig_spans])


    same_auth_spans = [(r[0],doc[r[0]:r[1]+1],True) for r in (ranges([i for i in range(len(pos)) if
pos[i] == note["NOTE_AUTH"]]))]
    same_auth_len_char = sum([len(s[1].text) for s in same_auth_spans])
    same_auth_len_tok = sum([len(s[1]) for s in same_auth_spans])


    cp_len_tok = sum([len(s[1]) for s in cp_spans + orig_spans if s[2]])
    cp_len_char = sum([len(s[1].text) for s in cp_spans + orig_spans if s[2]])

out.append({'cp_len_char':str(cp_len_char),'cp_len_tok':str(cp_len_tok),'tot_len_tok':str(total_len
gth_tok),'tot_len_char':str(total_length_char),'nid':nid,'same_auth_len_char':str(same_auth_len_
char),'same_auth_len_tok':str(same_auth_len_tok)})

  return out



# run the UDF on all notes
cp_function = F.udf(spark_cp_count,ArrayType(MapType(StringType(),StringType())))
cpdata = grouped.withColumn('text_length',cp_function(grouped.notes)).cache()
b = cpdata.select('text_length')
a = b.select(F.explode(b.text_length).alias('text_length'))

c =
a.select(F.col('text_length.nid').alias('NOTE_ID'),F.col('text_length.cp_len_tok').alias('cp_len_tok
'), F.col('text_length.tot_len_tok').alias('tot_len_tok'),
F.col('text_length.same_auth_len_tok').alias('same_auth_len_tok'),F.col('text_length.same_auth
```

© 2022 Steinkamp J et al. *JAMA Network Open.*

```
_len_char').alias('same_auth_len_char'),F.col('text_length.cp_len_char').alias('cp_len_char'),F.col('text_length.tot_len_char').alias('tot_len_char'))

combined = notes.join(c, ['NOTE_ID'],'inner')

combined.write.parquet(***OUTPUT FILE***)
```

© 2022 Steinkamp J et al. *JAMA Network Open.*

eAppendix 2. Comparison of Methodology to Prior Work

We deliberately chose to use the n-gram sliding window algorithm rather than the sequence alignment methods which have been used in prior work ([15,17]).

(1) We do not believe sequence alignment methods are well-suited to fully capture the scope of the clinical text duplication problem, for a few reasons. Traditional complete pairwise sequence alignment presumes that one sequence (note) is the result of copying and transforming exactly one other previous sequence (note). This has multiple problems; one is that duplication can occur at an intra-note level (e.g. text in the history/physical section of a note is pasted to the assessment/plan in the same note, etc). Also, clinical notes often include text duplicated from multiple sources (i.e., note C is made up of text duplicated from note A and note B). Using (naive) sequence alignment only allows for the alignment of a note text to a *single* other source which has been transformed, and does not capture the phenomenon of multiple sources. The n-gram method, by contrast, facilitates detection of these real-world phenomena more effectively.

(2) Performing pairwise sequence alignment on such a large corpus (even limiting the algorithm to only aligning a note with previous notes in the same patient's chart) would be prohibitively expensive computationally ($O(n^2)$ per word or worse). The n-gram algorithm, on the other hand, is $O(n)$ with respect to words, which is much more tractable. This is because we use a python dictionary (a data structure with a constant time lookup) to store all previously seen 10-grams, and each 10-gram in the document must simply be 'looked up' to see if it has been previously used in the patient's chart. Previous work that has used sequence alignment has either used much smaller corpora (Wrenn et. al.) or relied on assumptions about duplication that may not hold in reality. For instance, Rule "only computed redundancy for notes where the patient's last visit in a particular specialty had been with the same clinician and documented by the same

© 2022 Steinkamp J et al. *JAMA Network Open.*

author, providing a similarly constructed note for comparison. We did not compute redundancy for notes where the patient had no prior outpatient encounter in that particular specialty, or the most recent note had been written by another author.” We believe from clinical experience (and provide evidence in our study) that duplication from different authors is highly prevalent in the EMR, so our study requires an algorithm with the ability to compare sequences of word tokens to *all* prior sequences written for that patient. For these reasons, the n-gram algorithm is more suitable for our purposes.

One trade-off with using our algorithm instead of pairwise sequence alignment is that it may detect small segments of text which were not directly duplicated, but in fact just reflect common phrases used in clinical practice or templated text. This may lead to an overestimation of duplicate text content. To evaluate the scope of this problem, we conducted a preliminary analysis using 10-grams to identify how much text would be identified by our duplication detection *by chance* (i.e., without intentional duplication behavior) using this method. To do this, we selected 1,000 random notes from all patients in our corpus to use as a ‘randomly generated chart.’ We then selected a sample of notes from our corpus to treat as “new notes”, and ran the same duplication detection algorithm on the new notes, using the ‘randomly generated chart’ as the “previous notes” to compare to. In this way, we could identify how much text would appear “duplicated” based on the duplication caused by the fact that clinicians use certain turns of phrase, note templates, and text macros to document. We used a sample of 10,000 notes for this process (i.e., treat each of the 10,000 notes as if it was a “new note” written in the same chart as the “randomly generated chart”, and average the duplication metric across all text in these 10,000 notes). We found that 13.6% of text in a new note would be identified as duplicated by chance with a previous chart consisting of 1,000 notes, and 5.2% with a previous chart consisting of 100 notes. The vast majority of patients in our corpus have fewer than 1,000

© 2022 Steinkamp J et al. *JAMA Network Open.*

notes in their chart. On inspection, the “duplicated” text primarily consisted of standardized note templates, automatically generated medication lists and laboratory data.

© 2022 Steinkamp J et al. *JAMA Network Open.*

eAppendix 3. Discussion of Limitations of Our Methods

Our metric for identifying the "source" of duplicated text (i.e., the prior note that the current author was looking at or thinking about when they duplicated the text) is imperfect. Our metric identifies the source as the *most recently written note which includes the duplicated text,* which is an assumption that may not always hold in reality (the author may have duplicated the text from an older note, rather than the most recent).

However, any metric to identify the true source of duplicated text will be somewhat flawed without direct monitoring of the user's duplication actions, which is infeasible for a study of this size. Some EMR software, including Epic, includes functionality to track limited subsets of duplicate text; to do this, the EMR records when note text was copy-pasted from another note in the chart, as well as the source of the copy-pasted text. However, this functionality only captures directly copy-pasted text (e.g. with Ctrl+C / Ctrl-V) and excludes all other forms of duplication, including duplicate text generated by templates, users re-typing out the same text, or minor paraphrases which nonetheless contain large amounts of redundancy. We therefore felt this feature was too limited for our purposes.

For the purposes of this study, we only aim to claim that duplicate text is a problem that arises both from the individual user documenting repeatedly over time (sourcing from past notes they have written) as well as from multiple users duplicating each other's past work, which we believe our metric is sufficient to demonstrate. We believe our metric is sufficient to demonstrate the point that both inter-author and intra-author duplication are both relatively prominent contributors to text duplication.

On the other hand, there may also be interesting questions that result from defining the "source"

as the *first* appearance of the text chunk rather than the most recent (e.g. how many viral copies is the first instance of a 10-gram “responsible” for); we aim to evaluate these questions in a future study.

© 2022 Steinkamp J et al. *JAMA Network Open.*

eTable 1. Median, Mean, and Total Note Length in Characters by Note Type

Note Type	Total Characters (All Notes of Type)	Median Note Length (Characters)	Mean Note Length (Characters)
History & Physical (Inpatient)	3.46E+09	7030	7645
(Initial) Consult Note	4.85E+09	7028	7419
Medical Student Note	2.74E+08	5688	6351
Discharge Summary	3.33E+09	4800	5584
Progress Note (Inpatient)	2.16E+10	4214	4697
Nursing Assessment	7.9E+09	3724	4055
History & Physical (Outpatient)	2.22E+09	3273	4245
Operative Note	1.72E+09	2817	3038
Progress Note (Outpatient)	9.07E+10	2339	3581
Nursing Plan of Care	1E+10	1791	2533
Patient Instructions	1.19E+10	899	2887
Telephone Encounter	1.11E+10	160	294
Result Note*	1.72E+08	56	114

*Note used to document actions taken on laboratory results in the outpatient setting (in our system, the common practice is not to amend existing notes, but to create new notes for this purpose).

© 2022 Steinkamp J et al. *JAMA Network Open.*

eTable 2. Note Lengths by Provider Type, Sorted by Median Note Length

Provider Type	Total Characters (All Notes written by Provider Type)	Median Note Length (Characters)	Mean Note Length (Characters)
Occupational Therapist	2.21E+09	6580	5848
Physical Therapist	3.22E+09	5853	5323
Medical Student	7.78E+08	4153	4672
Resident	1.63E+10	2590	3775
Fellow	3.37E+09	2485	4072
Attending Physician	8.73E+10	1508	3129
Physician Assistant	9.16E+09	1504	3144
Pharmacist	9.98E+08	870	1259
Nurse Practitioner	2.45E+10	717	2921
Social Worker	1.39E+09	623	1094
Case Manager	9.62E+08	618	913
Registered Nurse	2.02E+10	207	787
Front Desk Staff	2.73E+09	151	261
Medical Assistant	4.68E+09	146	571

© 2022 Steinkamp J et al. *JAMA Network Open.*

eAppendix 4. The Utility of Shared Clinical Templates

Our study does not thoroughly evaluate the types or content of duplicate text (e.g. the prevalence of shared clinical templates vs. auto-generated content e.g. medication lists vs. deliberately typed duplicate free-text, etc.) A qualitative examination of a sample of the duplicated text would shed further light on these questions. In future work, we plan to perform this qualitative study.

However, it is important to note that our quantitative method for identifying duplicate text deliberately does not capture text duplication across two different patient charts (inter-patient duplication) - it only captures text duplicated within a single patient's chart (intra-patient duplication). This is intentional, as much inter-patient duplicate text reflects shared clinical templates. Shared clinical templates referring to different patients are in fact stating *different things.* For instance, a clinician may use a 'heart failure' template as part of an assessment/plan section in notes for both Patient A and Patient B. In the Patient A note, the clinician is stating that Patient A has heart failure and should be treated with a certain regimen; in the Patient B note, the clinician is stating the same of Patient B, which is not duplicate information. This may seem an obvious point, but it is important to recognize in the discussion around the utility or appropriateness of duplicating text.

According to our methodology, if a template appears in a single note for patient 1 and a single note for patient 2, neither is counted as duplicate. This is intentional, as this does not necessarily reflect undesirable behavior. Medicine is often rather stereotyped, so a physician who treats the same condition thousands of times may develop a templated set of diagnostics/treatments/patient instructions, and having this template available will save them significant time. Similarly, healthcare systems which seek to enforce high-quality treatment

© 2022 Steinkamp J et al. *JAMA Network Open.*

standards may develop templates for entire departments or organizations, which also makes sense and justifies ‘duplication’ across patients (which, again, is not counted as duplication by our method).

On the other hand, we do not believe there are many cases where it makes sense to document the exact same thing for the same patient multiple times (although it may be the only/best thing to do under the current ‘note’ paradigm). For instance, many notes for the same patient might contain the same history of present illness, list of medications, or in the case of progress notes, the same treatment plan copy-pasted multiple times across multiple days or multiple consultant teams’ notes. We believe these duplication instances actually reflect failures of the current documentation paradigm, because clinicians should not need to write out the entirety of the duplicated text again merely to state the relevant fact - that the *old information remains true at the current time.* Being able to state that old information remains true without writing it all out again (and building systems which facilitate this as the default) would be a significant improvement over the current paradigm which would likely significantly reduce duplication. These intra-patient duplications are the only duplications our method captures, by design.

© 2022 Steinkamp J et al. *JAMA Network Open.*
